# Efficacy of hyperbaric oxygen treatment for depression in the convalescent stage following cerebral hemorrhage

**DOI:** 10.3892/etm.2013.1043

**Published:** 2013-04-02

**Authors:** HUA CAO, KEJU JU, LINGLING ZHONG, TAO MENG

**Affiliations:** Department of Neurology, No. 1 People’s Hospital Affiliated to Nanjing University of Medical Sciences, Huaian, Jiangsu 223300, P.R. China

**Keywords:** cerebral hemorrhage, convalescent stage, hyperbaric oxygen, depression

## Abstract

The present study aimed to evaluate the clinical efficacy of hyperbaric oxygen (HBO) treatment for depression in the convalescent stage following cerebral hemorrhage. A total of 60 cases of patients with depression in the convalescent stage following cerebral hemorrhage (2–6 months) were randomly divided into the treatment group (treated with HBO, 30 cases) and the control group (treated with Deanxit, 30 cases). Prior to treatment and at 4 weeks post-treatment, efficacy was evaluated by the Hamilton Depression Scale (HAMD) and nerve function defect scores. There was a significant difference in the total efficacy between the two groups (P<0.05), and a significant difference in the HAMD scores (P<0.05). There were also significant differences between the pre- and post-treatment HAMD scores within the two groups (both P<0.05). HBO is able to significantly improve the degree of depression in the convalescent stage following cerebral hemorrhage and also promote nerve function recovery.

## Introduction

Depression is one of the most common mental disorders, with a lifetime incidence rate of 15–20% worldwide (1). This disease not only aggravates the cognitive impairment of patients, but also affects their quality of life, hinders the recovery of nerve function and markedly increases the mortality rate (2). The depression incidence rate exhibits a rising trend year by year, and refractory depression has consequently become a research hotspot for psychiatric medicine in recent years. Research into the etiology, pathogenesis and acology of refractory depression attracts widespread attention from scholars. At present, there are numerous drug types for treating depression, each with their own advantages. However, one drug does not appear to have a stronger effect than any of the other drugs, and only disease tolerance is marginally improved. Depression is highly recurrent and refractory depression is easily caused. Therefore, it is necessary to pay attention to the clinical standardized comprehensive treatment administered upon the first occurrence of depression, in order to stabilize and relieve the disease conditions and finally achieve an effective cure.

In the last 20 years, there has been great progress in the research on antidepressants, however 15–33% of depression patients have been identified as insensitive to drug treatments (3). Once treatments of two or more antidepressants with different chemical structures have been administered in an adequate dose and course, refractory depression occurs if the treatment efficacy is only small or non-existent (3). Certain scholars (4) have reported that following acute cerebral apoplexy, the post-stroke depression (PSD) incidence rate was 20–39% and the PSD incidence rate among elderly individuals reached 50–60% (4,5). Depression following cerebral hemorrhage is more marked in the convalescent stage. The depression incidence rate is higher, particularly 6 months subsequent to the cerebral hemorrhage. There are more studies on depression following cerebral infarction and fewer studies on depression following cerebral hemorrhage. The depression incidence rate of patients with cerebral hemorrhage is closely corelated with the bleeding site and the extent of cognitive impairment in nerve function (6). Cephaledema is the main pathophysiological change following cerebral hemorrhage and a significant measure for relieving degrees of secondary brain injury and protecting and promoting nerve function recovery to effectively treat encephaledema (7). Previously, scholars have conducted wider research into hyperbaric oxygen (HBO) treatment of cerebral hemorrhages and confirmed that HBO had a definite, recordable efficacy (8). In the present study, auxiliary HBO treatment was used in the convalescent stage and satisfactory efficacy results were recorded.

## Subjects and methods

### Clinical data

A total of 200 patients with hypertensive cerebral hemorrhage who received treatment in the Department of Neurology (No. 1 People’s Hospital Affiliated to Nanjing University of Medical Sciences, Jiangsu, China) between January 2009 and October 2011 were selected. The measures used to treat their symptoms included dehydration to reduce the intracranial pressure, the use of a neural activation agent, nutritional maintenance and the minimally invasive removal of brain hematomas. All patients were suffering from the first occurrence of cerebral hemorrhage. There were 121 male and 79 female cases, with a mean age of 57.2±14.3 years and disease courses ranging from 2–6 months. All cases complied with the diagnostic code for cerebral hemorrhage prepared by the Fourth National Conference on Cerebrovascular Disease in 1996. Disorders of consciousness and complete sensory aphasia or anandia were excluded, while dementia or mental symptoms were included. A depression assessment was conducted using the Hamilton Depression Scale (HAMD) and nerve function defect scoring was conducted using the National Institutes of Health Stroke Scale (NIHSS) 2–6 months subsequent to onset. A HAMD score of >20 points was regarded as the judgment criterion for depression. A total of 60 patients with depression were divided into two groups: the HBO group (30 cases) and the control group (30 cases). In the HBO group, there were 11 male and 19 female cases, with a mean age of 57±13 years. In the control group, there were 12 male and 18 female cases, with a mean age of 56±17 years. When considering age, gender, depression severity and time from cerebral hemorrhage onset to inclusion treatment, there were significant differences between the two groups (P<0.05). The present study was conducted in accordance with the declaration of Helsinki and with approval from the Ethics Committee of the No. 1 People’s Hospital Affiliated to the Nanjing University of Medical Sciences. Written informed consent was obtained from all participants.

### Follow-up

One month subsequent to onset was defined as the acute stage of cerebral infarction and the period from 2–6 months subsequent to onset was defined as the convalescent stage of cerebral infarction. The depression conditions of the patients at 2–6 months subsequent to onset were evaluated. The follow-up was carried out by home or outpatient return visits.

### HBO treatment

In the acute stage of cerebral infarction, conventional therapy and general psychotherapy were conducted on the two groups in the internal neurological department. The HBO group were treated in a multiple-person, large HBO cabin with a pressure of 0.2 MPa (2 ATA). Dexamethasone (2.5–5 mg) was conventionally administered 10–20 min prior to entering the cabin. Subsequent to entering, pure oxygen was breathed in via a face mask or head mask twice for 35 min/time and the air in the cabin was breathed in for 10 min in between. At the same time, sputum suction was conducted in the cabin to ensure that the respiratory tract remained unobstructed, while electrocardiogram monitoring was performed outside of the cabin. In any instances of change to the disease condition, treatment was immediately provided. A single treatment course included 10 treatment sessions and a total of 3 treatment courses were conducted. In the control group, 2 Deanxit tablets (0.5 mg depixol and 10 mg melitracen; Denmark Lingbei Pharmaceutical Company, SFDA Approval Word H20020474; compound preparation) were administered daily, one in the morning and one at noon. Following an evident improvement in symptoms only one tablet was administered in the morning. The treatment courses of the two groups lasted 4 weeks. Prior to treatment and at 4 weeks post-treatment, the treatment efficacy was evaluated according to the HAMD score. In addition, routine blood, urine, liver and kidney functions and electrocardiographic examinations were conducted prior to treatment and at 4 weeks post-treatment. Adverse reactions were also recorded.

### Judgment of clinical efficacy

The clinical efficacy was determined once prior to treatment and once at 4 weeks post-treatment. According to the HAMD score reduction rate standard, a score reduction rate of ≥75% represented a cure, ≥50% represented marked progress, ≥25% represented progress and <25% represented a fail.

### Statistical analysis

SPSS 10.0 software was used for the statistical analyses, with the t-test and χ^2^ test performed to examine the differences in the data between the groups. P<0.05 was considered to indicate a statistically significant difference.

## Results

### NIHSS and HAMD scores

The NIHSS and HAMD scores showed significant differences between the pre-treatment and post-treatment results within each group (P<0.01); the NIHSS and HAMD scores also showed significant differences between the control and treatment groups post-treatment (P<0.05). The results are shown in Table I.

### Clinical efficacy comparison

The efficacy to secondary depressive disorder following cerebral hemorrhage was observed and evaluated by the HAMD score. The score reduction rates of the HBO and Deanxit control groups were 83.3 and 60.0%, respectively, at 4 weeks post-treatment and demonstrated a statistically significant difference (P<0.05, Table II).

## Discussion

PSD involves a set of comprehensive clinical syndromes complicated by cerebral apoplexy. The incidence rate of PSD is high and may reach 30–50% of patients with acute cerebral apoplexy (9). One month subsequent to onset is defined as the acute stage of cerebral hemorrhage and the period from 2–6 months subsequent to onset is defined as the convalescent stage of cerebral hemorrhage. It is possible that certain composite factors, including a decrease in social contact or loss of social activities, poor family relationships, living alone, poor social support and poor psychological coping abilities subsequent to onset cause patients to suffer depression. PSD occurrence and extent directly affect recovery of paralytic limb function and nerve function defects and the quality of life of patients with cerebral apoplexy, as well as the cerebrovascular disease mortality rate (10,11). Cerebral hypoxic ischemic injury possibly affects the brainstem, thalamus, basal ganglia, cortex of frontal lobe and other areas to cause depression (12,13). Therefore, an improvement in the oxygen supply to the brain in patients with strokes is not only able to reduce the secondary damage to the cerebral cortex and relevant nerve functions, but is also able to promote brain remodeling and functional reorganization in patients with cerebral apoplexy in the convalescence stage. Scholars have hypothesized that the onset of depression following cerebral hemorrhage is possibly associated with the dysequilibrium of noradrenaline and 5-hydroxytryptamine caused by cerebral lesions (14,15). Damage to nerve functions seriously affects the daily abilities of patients and limits their ability to have a social life, thus causing a series of psychological symptoms. In the present study, HBO was used to treat depression in the convalescent stage following cerebral hemorrhage and a good result was obtained. HBO therapy maintains the body in a high atmospheric pressure environment (>1 atm) and allows the patient to breathe pure or mixed HBO (97% O_2_: +>3% CO_2_:) at the same pressure as the environment. HBO is successfully used to treat various diseases (16). The present study suggested that in the 30 cases receiving HBO treatment, the depressed mood of the patients was improved, the nerve function defect score was markedly reduced, cognitive ability and movement functions were enhanced and the NIHSS score improved from 19.12 pre-treatment to 8.4±2.9 post-treatment. Also, the HAMD score dropped from 30.4±13.6 pre-treatment to 9.4±5.4 post-treatment, with a significant difference between the pre- and post-treatment results. Compared with the simple anxiolytic control group that were only administered Deanxit, there was a significant difference in the nerve function recovery scores of the HBO group and the efficacy was improved. Significantly, there was also no evident adverse reaction. Therefore, HBO treatment is worthy of further promotion and application in the clinic. The depression symptoms that appear subsequent to cerebral hemorrhage are correlated with the patient’s central nervous injuries (6). Following cerebral hemorrhage, depression in the convalescent stage is more evident. The depression incidence rate is higher, particularly 6 months subsequent to hemorrhage. Depression in the convalescent stage is a common effect of physiological and psychological factors (17) and greatly affects patient recovery. Depression may increase nerve function defects, delay the recovery process, increase medical costs and even cause certain patients to commit suicide, resulting in a great loss to their family. In cases of PSD, active treatments are required. Currently, the consensus that early antidepressant treatments and timely eradication of the emotional disorder is necessary to promote the recovery of nerve functions has been reached worldwide. In addition to drug treatment alone, there are a range of drug treatments combined with psychological and electroconvulsive therapies, as well as other methods. With the development of bio-psycho-social medical models, depression following cerebral hemorrhage should be treated from multiple, overall and comprehensive viewpoints to intervene in the advancement of the condition and form a diagnosis and treatment as early as possible. When using HBO, the vertebral artery blood flow rises (by 18% under 0.2 MPa O_2_) (18), which causes an increase in the partial pressure of oxygen in the formatic reticularis and brainstem and effectively improves the internal inhibition weakening phenomenon of the cerebral cortex. An increased partial pressure of oxygen also enhances the regulation and control of the subcortical autonomic nervous system and promotes rehabilitation. When using HBO, energy generation is increased, therefore, HBO forces the brain tissue to obtain sufficient oxygen to increase its oxygen metabolism rate, enhance glucose availability and promote the recovery of dysregulated cortical functions. In addition, HBO is able to elevate arterial oxygen saturation, increase blood oxygen content, improve the extent of atherosclerosis, promote repair of vascular lesions and regulate the central nervous system. HBO is also able to increase the activity of the neuronal cell pentose phosphate pathway to promote neuronal metabolism recovery.

The results of the present study show that for patients with depression in the convalescent stage following cerebral hemorrhage, the total efficacy of the HBO treatment (conducted additionally to the conventional treatment) is higher than that of the control treatment. At 1 month post-treatment, the NIHSS and HAMD scores of the treatment group significantly decreased and there was a significant difference identified between the treatment and control groups. These results suggest that HBO aids the recovery of nerve function and improves depression symptoms. The importance of specific steps have also been highlighted in the present study. For example, it is necessary to ensure that the patients adequately breathe in the oxygen while in the HBO cabin. Also, for patients with excess secretions in the respiratory tract, it is necessary to perform sputum suction to ensure that the respiratory tract remains unobstructed and to conduct electrocardiogram monitoring outside of the cabin to observe any changes to vital signs and treat problems as they occur. Lastly, the treatment process may not be interrupted. HBO therapy consists of one treatment course including 10 treatment sessions, with a minimum of three treatment courses required to improve the efficacy. As the number number of cases in the present study was small, this viewpoint requires further confirmation. HBO therapy may also have further potential for use in the treatment of depression in the acute phase following cerebral hemorrhage.

## Figures and Tables

**Table I:** Comparisons of the NIHSS and HAMD scores between the two groups.

	NIHSS		HAMD	
	
Groups	Pre-treatment	Post-treatment	Pre-treatment	Post-treatment
Hyperbaric oxygen	19.2±3.9	8.4±2.9	30.4±13.6	9.4±5.4
Control	19.7±3.7	13.2±4.1	30.7±14.5	19.7±4.4

Results are presented as mean ± SD. Comparison between the two groups pre-treatment, P<0.01; and post-treatment, P<0.05. NIHSS, National Institutes of Health Stroke Scale; HAMD, Hamilton Depression Scale.

**Table II:** Comparison of clinical efficacy.

Groups	n	Cure	Marked progress	Progress	Fail	Total efficacy (%)
Hyperbaric oxygen	30	12	7	6	5	83.3
Control	30	4	6	8	12	60.0

Comparison between the two groups post-treatment, P<0.05.
